# Using Social Media to Understand Web-Based Social Factors Concerning Obesity: Systematic Review

**DOI:** 10.2196/25552

**Published:** 2022-03-07

**Authors:** Chuqin Li, Adesoji Ademiluyi, Yaorong Ge, Albert Park

**Affiliations:** 1 University of North Carolina at Charlotte Charlotte, NC United States

**Keywords:** obesity, web-based social factors, systematic review, social-ecological model

## Abstract

**Background:**

Evidence in the literature surrounding obesity suggests that social factors play a substantial role in the spread of obesity. Although social ties with a friend who is obese increase the probability of becoming obese, the role of social media in this dynamic remains underexplored in obesity research. Given the rapid proliferation of social media in recent years, individuals socialize through social media and share their health-related daily routines, including dieting and exercising. Thus, it is timely and imperative to review previous studies focused on social factors in social media and obesity.

**Objective:**

This study aims to examine web-based social factors in relation to obesity research.

**Methods:**

We conducted a systematic review. We searched PubMed, Association for Computing Machinery, and ScienceDirect for articles published by July 5, 2019. Web-based social factors that are related to obesity behaviors were studied and analyzed.

**Results:**

In total, 1608 studies were identified from the selected databases. Of these 1608 studies, 50 (3.11%) studies met the eligibility criteria. In total, 10 types of web-based social factors were identified, and a socioecological model was adopted to explain their potential impact on an individual from varying levels of web-based social structure to social media users’ connection to the real world.

**Conclusions:**

We found 4 levels of interaction in social media. Gender was the only factor found at the individual level, and it affects user’s web-based obesity-related behaviors. Social support was the predominant factor identified, which benefits users in their weight loss journey at the interpersonal level. Some factors, such as stigma were also found to be associated with a healthy web-based social environment. Understanding the effectiveness of these factors is essential to help users create and maintain a healthy lifestyle.

## Introduction

### Background

The obesity epidemic is a significant public health challenge in modern society. The growing prevalence of obesity and its implications in public health make it one of the most common, dangerous, and costly diseases globally [1]. One-third of the global population, over 2 billion people, are overweight or obese [2]. Obesity rates reached 39.8% among adults and 18.5% among youth in the United States in 2016, a significant increase in these age groups since 1999 [3].

Obesity is recognized as a major risk factor for population health [4] because of its association with social stigma [5], chronic diseases [6], medical complications [7], reduced life expectancy [8], lower quality of life [9], and higher health costs for individuals [10] and the government [11]. The World Health Organization suggests that obesity is likely the cause of chronic diseases such as hypertension, type 2 diabetes, cardiovascular diseases, and some cancers [12]. Women with obesity are more vulnerable to infertility, miscarriage, and other childbearing-related complications [7]. Obesity in childhood also increases the risk of other diseases and may even be carried through adolescence to adulthood [6]. Obesity also increases individual and fiscal expenditure. The annual medical cost of obesity in the United States was US $147 billion in 2008, and the annual medical cost for an individual with obesity was estimated to be US $1429 higher than that for individuals with a healthy weight [11]. Obesity may affect human development [8], suggesting that its prevalence could be detrimental to human life expectancy in the 21st century, reversing the increase in life expectancy seen in the 20th century. Global efforts are paramount in controlling the obesity pandemic.

### Social Factors as Important Drivers of Obesity Pandemic

Recent developments in research have identified two main factors—exercise and diet [13,14]; however, there are other factors associated with obesity [15]. According to a study, a developed society is the leading cause of the current obesity pandemic in that it creates an obesogenic environment [16]. An obesogenic environment is defined as an environment where there is easy access to inexpensive and calorie-dense food, excessive food intake, insufficient physical activity, and inexpensive nonphysical entertainment [7,17]. An obesity epidemic with interconnected social factors could result in an obesity pandemic.

Social factors are defined as factors that affect an individual’s lifestyle [18]. These influences have a significant effect on people’s health-related behaviors [13,19]. Thus, social factors play an important role in the spread of obesity. For example, a study conducted by Christakis and Fowler [20] tracked a densely interconnected social network of 12,067 people for 32 years. It showed that a person's chances of becoming obese increases by 57% if he or she has a close relationship with someone who is obese. Furthermore, the self-perception of weight can also be influenced by peers. Previous research indicates that children and adolescents who are surrounded by many overweight peers might have inaccurate perceptions of their weight and underestimate it [21,22].

With the recent ubiquity of social technologies, these peer effects are expanding to the general public. A recent study on a large-scale social network showed that social influences also affect collective public health behaviors, such as habits associated with obesity and tobacco use [23]. Similarly, examining user interactions on social media has proven useful in understanding public attitudes and perceptions of health topics [24]. As a result, it is timely and imperative to understand web-based social factors in a web-based social environment to counteract the obesity epidemic.

### Social Media for Understanding Obesity

Social network websites serve as web-based services that allow individuals to build social profiles, form connections with other users, and view other profiles in the system [25]. Popular social network websites such as Facebook, Twitter, and Reddit have attracted millions of users since they were first introduced in 2004, 2006, and 2005, respectively. A 2018 survey of US adults found that the social media landscape shows a long-standing trend of continuous use throughout the day and newly emerging narratives (eg, posts, tweets, and images) [26]. For example, 69% of US adults use at least one social media website; 74% of Facebook users and 46% of Twitter users access the website daily [27]. Internet integration may offer possibilities for accessing obesity-related information, including weight loss, obesity diagnosis, and weight management.

Social media platforms have the potential to change users’ health behaviors. An increasing number of users' social interactions are publicly shared on the web, making social media a vital data source for studying public health, especially *lifestyle diseases* such as obesity [24]. Chang et al [28] systematically reviewed the use and impact of social media in web-based weight management and demonstrated that social media plays a role in retaining and engaging participants in weight management.

### Review Aim

The primary aim of this review is to extend the knowledge on the influences of web-based social factors concerning obesity-related behavior to better inform future studies in examining interventions using social factors. This is the first study to systematically review the effects of web-based social factors on obesity-related behaviors in the web-based social media environment. Other related systematic reviews have examined obesity, social media use, and the effectiveness of social media in intervention studies for obesity prevention [29,30].

## Methods

### Data Sources and Search Strategy

We used three popular electronic databases—PubMed, Association for Computing Machinery (ACM), and ScienceDirect—in this review. PubMed is known as a comprehensive database in biomedical research [31]; the ACM database is maintained by the world's largest scientific and educational computing society [32]; and ScienceDirect provides access to an extensive database of scientific and medical research [33]. The search strategy in ACM and ScienceDirect was designed by combining the search terms social media and obesity. The full search string in ACM and ScienceDirect was *Social media* AND *obesity*. The MeSH (Medical Subject Heading) terms *social media* [34] and *obesity* were used in the PubMed search. The full search string in PubMed was *Social Media* (MeSH) AND *Obesity* (MeSH). All searches were completed on July 5, 2019.

### Study Selection and Screening

This study aims to review studies that used social media with elements of social factors for obesity research. To meet the review aim, we defined social media in this study as an internet-based platform allowing individual users to create and exchange content (eg, blogs, web-based discussion boards, and Twitter) based on a previous study by Kaplan and Haenlein [35].

All studies that met the inclusion criteria were included in this review. Inclusion criteria were defined as follows: (1) obesity was the primary study topic, (2) social media served as the main platform, (3) social interactions were incorporated, (4) the study was published in the peer-reviewed literature, and (5) the study was in the English language. To understand how web-based social factors can influence people in understanding or improving weight management outcomes, we either set organic or encouraged social interaction as an inclusion criterion. Other types of scholarly articles were excluded: comments, systematic reviews, conference reports, and letters. Moreover, design studies that only suggested the use of social media, such as a randomized controlled trial study design by Willis et al [36], were excluded.

On the basis of title and abstract, 2 independent reviewers first screened all articles. All articles were categorized into (1) included, if the paper met the inclusion criteria, (2) excluded, if this paper did not meet the inclusion criteria, and (3) needed full-text review, if the abstract could not provide enough information or was not available. A paper was then excluded at the screening stage if the 2 reviewers agreed to exclude it based on title and abstract. Except for the excluded articles, all articles were moved to the eligibility stage, which required 2 reviewers to perform a full-text review. At the eligibility stage, any disagreement was discussed to form a consensus. The third reviewer, a tiebreaker, was introduced if consensus could not be reached.

## Results

### Overview

**Figure 1:**
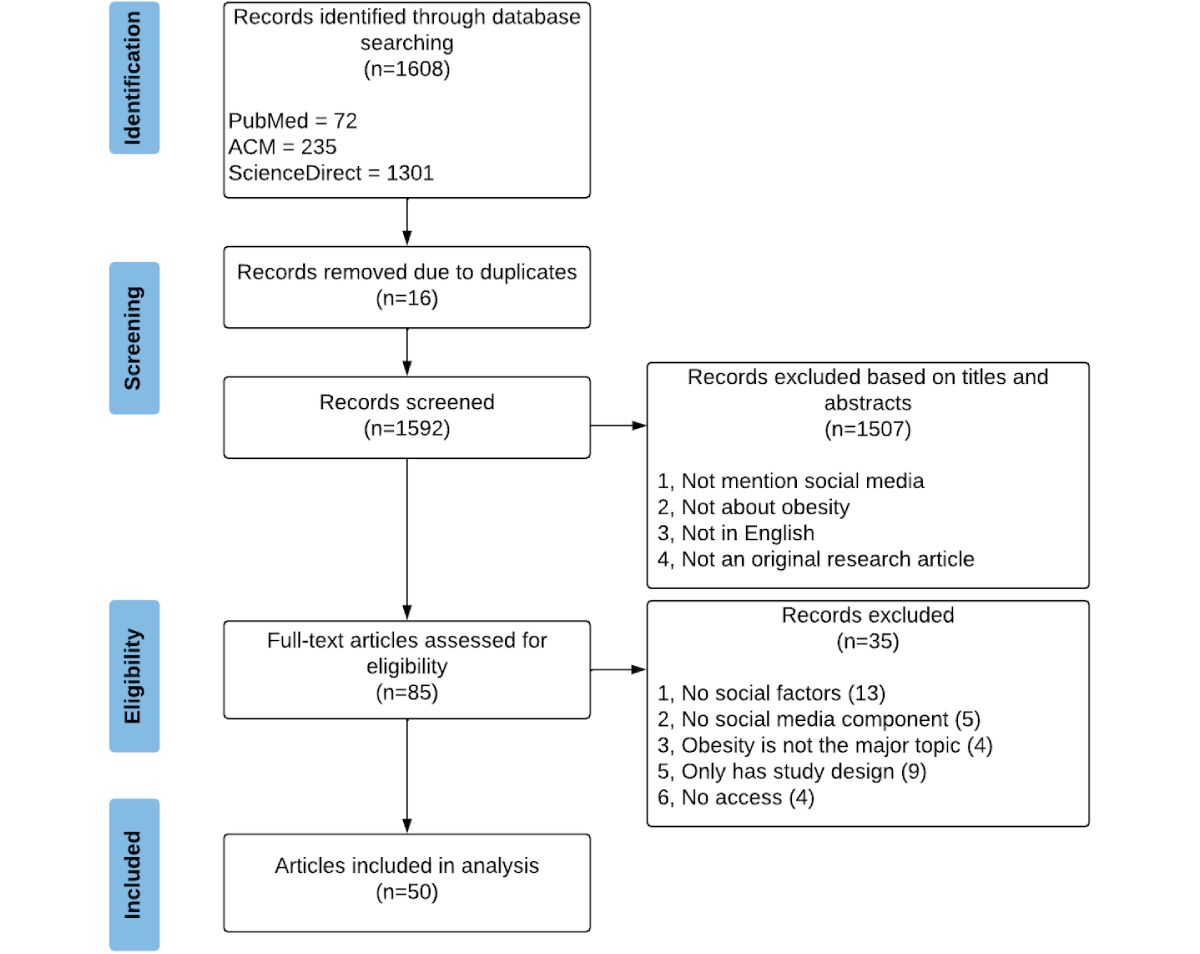
Study flow.

In total, 1608 studies were identified from our selected databases and search strategy, of which 16 (1%) were duplicated (Figure 1). After removing the duplicates and assessing the title and abstract, 93.72% (1507/1608) of the articles were excluded, and 5.29% (85/1608) remained for full-text reading. A full-text examination excluded 2.18% (35/1608) of the articles. In total, 3.11% (50/1608) of the articles met our inclusion criteria and were included in this systematic review. We summarized the internet-based social factors and their corresponding effectiveness in the following section. Furthermore, we examined how different social media platforms were used in previous studies.

### Web-Based Social Factors

#### Overview

Traditionally, obesity is linked to behavior at the individual level, such as overeating and lack of exercise. However, new studies have shed light on social factors that contribute to obesity-related behaviors. We investigated web-based social factors in this study. There is no universal definition of web-based social factors in the literature. Here, we defined web-based social factors as social factors that exist in web-based social environments and have the potential to affect users’ behaviors. We focused on identifying web-based social factors and understanding their potential effect on users’ obesity-related behaviors. We found 10 different web-based social factors used and mentioned in previous studies. The most common to the least common web-based social factors were social support and social ties, gender, geo-cultural factors, stigma, obesogenic environment, source credibility, school environments, social movements, policy, and social sharing behaviors. We will first discuss the most frequently mentioned social factors, social support and social ties.

#### Social Support and Social Ties

Social support is emotional comfort and material resources provided by peers connected in a social network. It is the most frequently mentioned factor in previous studies related to our study aim. Users of social media platforms can exchange social support. They view social media as a good place for finding and receiving social support and locating information platforms for those interested in changing their lifestyle and eating habits [37]. A previous study found that users who tried to use Twitter to record their weight loss journey reported receiving more social support from the internet-based environment than from their real families and friends [38].

In web-based communities, we identified two types of social support: informational support and emotional support. Informational support includes sharing resources and providing professional feedback through social networks [39-41]. Emotional support primarily comes from peers. Social culture and the concept of social media encourage users to be active with other users. In some intervention studies [40], program participants were instructed to discuss progress, issues, and goals with other participants using social media platforms. Through various platforms or programs, peer encouragement [42], peer support [43,44], and peer pressure [41] were identified.

Moreover, two platforms were found to be the major conduits for social support: blogs and Facebook. Savolainen [45] asserted that the main strength of web-based blogs is that they can provide emotionally supportive forums for sharing opinions. From a blogger’s perspective, Leggatt-Cook and Chamberlain [43] learned that bloggers hope to create and build a community that will support them and their attempt to lose weight. Facebook’s private groups have been widely used in intervention studies. These groups were created to share resources and serve as a platform for participants to communicate [40]. In a study by Waring et al [41], first-time mothers were often found to seek out other mothers’ advice and support from Facebook groups. Twitter is primarily used to collect public opinions, but it was also found to offer the opportunity to create a supportive network [39].

Social support is suggested to be very important for users who try to lose weight. Lack of support had a negative effect on weight loss. Pappa et al [44] found that the peer search for support was inversely associated with weight loss. Even in the anonymous platform Reddit, the authors observed an increasing number of users returning to the community, and greater weight loss was reported from users if they received support in the community [42]. The effectiveness of social support in weight loss has been reported to be positive. He et al [46] found that social support positively correlated with weight loss. Jane et al [37] also found that individuals had better health outcomes if supported by professionals. Chomutare et al [47] mentioned that their study found a positive correlation between web-based participation and weight loss by analyzing data on older women with obesity who were active in a web-based community. A study conducted on individuals with mental illness also found that weight loss was associated with perceived peer-group support because the participants felt compelled to pursue weight loss goals [48]. We also found a detracting study on the influence of social support on BMI reduction. An experiment on college students showed that students in the Facebook support group did not show a significant difference in BMI compared with the control group at the end of 24 months. However, the author reported that students in the intervention arm showed a significantly greater increase in the number of appropriate weight control strategies than those students who were not in the support group [49]. Similarly, a meta-analysis by Merchant et al [40] suggested that interventions that provide participants with professional support during their diets and physical activities are more effective than those that do not.

Social support is understood through social ties, the connections among peers. The social tie theory concludes that the probability of a person becoming obese increases if friends with obesity surround them [20]. A study by Phan et al [50] adopted the social tie theory in their experimental study, confirming that individuals tend to perform similar lifestyle behaviors as their friends from the internet-based environment. Social ties also affect received social support. Social support has a negative correlation with weak ties (ie, unfamiliar individuals) in the context of weight control. Chen et al [51] reported that participants found community competition and support from strong ties (eg, couples and parents) were motivating, whereas support from unfamiliar participants was demotivating.

#### Gender

We identified gender as a web-based social factor because we found 5 previous studies [39,41,52-54] indicating that women and men have different web-based social behaviors regarding obesity. Abbar et al [52] showed that women are often more willing to share information on the web, such as preparing low-calorie food, which was also supported by other related studies [55,56]. Women were more likely to share their family members’ weight management experiences on social media. Only mothers of a childhood weight loss camp were willing to use social media to receive informational support and post their children’s progress [39]. In a Twitter-delivered weight loss program, Waring et al [41] found that a great proportion of women read each other’s progress. These participants were reading other people’s tweets more than posting their own progress. Women were critical when self-evaluating their weights. In a study by Kuebler et al [53], Yahoo! Answers data found that most women, when asked about their self-perspective on their weight, tended to overestimate. Web-based social norms show the characteristics of women and men that are socially constructed.

#### Geo-Cultural Factors

Users’ health behaviors occur in a setting composed of web-based, social, and cultural environments. Geo-cultural factors explain how users’ behaviors on the web are affected by their physical surroundings. Several studies have found that social media data can provide insight into the health conditions of US residents. A total of 3 studies by Gore et al [57], Culotta [58], and Abbar et al [52] used Twitter data to predict county-level health. Abbar et al [52] discovered that the calories of food mentioned in tweets correlated to the county’s obesity rate. Gore et al [57] found that the tweets in areas with lower obesity rates had three features: (1) tweets had more positive sentiments, (2) more tweets mentioned fruits and vegetables, and (3) physical activities of any intensity were more frequently mentioned. Culotta [58] reported similar findings that negative emotions were found in tweets from areas with high obesity rates. Garimella [59] further validated the feasibility of using image data to track public health concerns. They found that user- and machine-generated image tags on Instagram could be used to forecast the county’s obesity rate. By analyzing pictures on Instagram, Mejova et al [60] found that the number of fast-food restaurants in a county in the United States positively correlated with local obesity rates. They further revealed that locally owned restaurants with dietary and nutritional alternatives were more popular in areas with lower obesity rates. Another interesting finding from Weber and Mejova [61] showed that the percentage of profiles with a valid profile picture was higher in areas with a higher obesity rate. Another branch of the geo-cultural factor is involved in culture and religion. After a weight loss camp in Qatar, a study concluded that the religious month and cultural orientation were critical to the outcome, affecting users’ web-based recording behavior [54]. These findings suggest that users’ web-based obesity-related behaviors are related to location-specific environments.

#### Stigma

Stigmatization and associated discrimination—sometimes referred to as weight bias—affect the individual’s mental and physical health and social behavior. Studies on Western culture highlight the stigmatization of individuals with obesity, showing that they are stigmatized and associated with laziness, low self-control, and moral laxity [55]. Social media is used to propagate social stigmatism, mainly in the form of fat-shaming, a practice of humiliating and criticizing overweight individuals on social media [62]. Mejova et al [62] found that up to 27.6% of non-URL tweets mentioning obesity were fat-shaming, with some self-hate messages. In a recent study by Karami et al [63], Twitter users often coupled exercise-related terms with obesity. This could also indicate that individuals associate exercise and self-regulation (or lack of it) as the main cause and solution for obesity. Although the social stigmatism of obesity is widespread on the web, individuals have also pushed back social stigmatism using social media platforms. People expressed anger caused by the stigma of obesity on Twitter in a retaliatory manner to address widespread stigmatization against overweight and obese people [64].

Stigma has been found to undermine a user’s mental health, but its effects on users’ web-based interactions remain inconclusive. A person’s mental health status revealed by social media data indicates that a user’s mental health is affected by their social surroundings. A study by Kuebler et al [53] suggested that people with obesity residing in counties with higher levels of BMI have better physical and mental health than people with obesity living in regions with a low obesity rate. Another 2 studies investigated the impact of weight stigma by comparing web-based behavior between users with normal weight and users with obesity. May et al [65] did not find weight bias in their study because weight status had no effect on the rate of interactions and follow backs. However, a different study by Weber et al [61] found that users who were labeled as overweight had fewer followers and fewer directed tweets. Although stigmatization may not affect the user’s web-based behavior, the widespread stigmatization on social media will diminish a user’s mental health.

#### Obesogenic Environment

An obesogenic environment refers to an environment that promotes high-energy intake and sedentary behavior [66]. By analyzing the content users post on social media, we found that an obesogenic environment is one of the major causes of the obesity pandemic. A content analysis of frequent retweets about obesity by So et al [64] revealed that four major social determinants of obesity are discussed on Twitter: cheap and unhealthy food, school food systems, portion sizes, and dysfunctional food systems. Among these determinants, easy access to cheap, calorie-dense foods had the highest tweeting rates. This finding suggests that the web-based information environment is changed by the physical obesogenic environment by informing users’ behaviors.

#### Source Credibility

Credible health information sources are persuasive [67]; however, some social media obesity-related information was found to be incomplete or inaccurate. A low-credibility source could exert a negative influence on users’ obesity-related behaviors. The primary reason revealed by previous studies is that information from professionals is lacking. Web-based information from professionals about obesity proved to be more accurate than that from other users. Erdem and Sisik [68] analyzed the content of 300 YouTube clips on bariatric surgery, also known as weight loss surgery, and suggested that the content from professional accounts tends to be more accurate. In another study, Basch et al [69] analyzed the top 100 most widely viewed weight loss videos on YouTube and found only 1 professional video; consumer-created videos dominated the domain. Mejova [62] examined 1.5 million tweets mentioning obesity and diabetes and found that only 23% of the content came from verified users (ie, Twitter accounts that are associated with a governmental or academic institution). Similarly, more individuals than organizations tweeted about childhood obesity [67].

Misinformation in content can also harm users. YouTube advertisements for rapid weight loss products and commercial videos focused too much on workouts instead of maintaining a balanced diet [69]. The top-cited domains relating to obesity and diabetes on Twitter are not affiliated with guidelines provided by governmental or academic institutions [24]. The discrepant information from user-generated content can lead to a drop in trust for these platforms. Messages presented in traditional social media platforms, such as blogs, were seen as a more reliable source than other newer social media platforms, such as Facebook [70]. Meitz et al [70] compared the source credibility perceptions among different platforms and found that messages on Facebook were perceived as significantly less relevant than messages presented in blogs. Together, these studies reinforce the importance of source credibility in conducting users’ health behaviors.

#### School Environment

School, serving as a center of childhood development, has an influential role in a child’s early behavioral development. Findings from social media content analysis suggest that the school environment is essential in affecting children’s obesity-related behaviors. From one aspect, the school decides students’ daily routines and food selection. So et al [64] argued against excess homework, and Harris et al [67] noted that most public schools do not regulate access to junk food. In addition, teachers’ participation in combating childhood obesity is critical. In a preschool obesity prevention curriculum, parents showed a strong desire for more engagement from their classroom teachers [71]. Changing the school environment was the most common strategy to combat childhood obesity mentioned on Twitter. For example, a person tweeted, “Americans expect schools to lead in preventing childhood obesity” [67]. Users’ attitudes toward school environments shape the internet-based discourse on childhood obesity.

#### Social Movements

Social movements are defined as organized efforts by a group of people to bring out or impede social or cultural changes [72]. Social media offers a new possibility of exploring social movements efficiently. In recent years, several distinct web-based trends regarding obesity have been found in social media: *body positivity*, *thinspiration*, *fitspiration*, and *HAES* (health at every size). The term *body positive* originally came from the 1960s feminist movement and resurfaced in the fat acceptance movement. A content analysis of *body-positive* images on Instagram showed that this movement seeks to challenge beauty standards while rejecting an inaccessible body image and promoting acceptance of all body types and appearances [55]. However, another prevailing trend called *thinspiration* surfaced with the intent of spreading thin body imagery and inspiring weight loss. Content analyses showed that body-positive images on Instagram drew a broad range of body sizes [55], whereas thinspiration images on Twitter tend to depict ultrathin and scantily clad women [73]. Exposure to guilt-inducing and body-objectifying messages has been found to increase body dissatisfaction and negative mood. The study also showed that the more times individuals view thinspiration content, the higher the probability they will report eating disorder symptoms [69]. Subsequently, a new trend called *Fatspiration*, supporting fat acceptance, has become prominent in the mainstream [56] and was found on the web. Another trend, *HAES*, promoting wellness rather than weight loss, was also identified. However, discrimination against obesity has not yet been resolved. Fat stigmatization content within the *HAES* and *Fatspiration* tags were found in a content analysis of Instagram images [74]. Social media shapes the web-based information environment and helps us fully understand the context of social movements. Without understanding the full scope, there is a potential for a negative shift in social norms.

#### Policy

Governments have tried to combat obesity by establishing new policies. Three studies [67,75,76] used social media to study the public’s attitude and reaction toward government policy. In 2016, the United Kingdom also published a plan to reduce England’s rate of childhood obesity within the next 10 years, *Childhood obesity: a plan for action*. Most comments to the related web-based newspaper articles were considered negative in a study by Gregg et al [76]. Later, in 2017, Kang et al [75] collected relevant tweets to investigate the public’s opinion on a new school meal policy. They found that 70% of tweets were neutral, although the number of negative tweets was still higher than that of positive tweets. Negative tweets expressed interest and concern about the policy and suspicion of the effectiveness of the campaign. Instead of worrying, some users also used social media to support the announcement and execution of policies. Harris et al [67] found *Bye Fitspiration Junk Food*, a US Department of Agriculture rule that requires healthier snacks for children and the adoption of the physical education classes as a core subject in schools, was a prominent movement in communication about childhood obesity on Twitter. Web-based information can shape an individual’s attitude toward a certain policy. With social media, policy makers can better disseminate policies and raise public awareness.

#### Social Sharing Behaviors

We found 2 psychology theories related to web-based sharing behaviors in the literature: social sharing of emotions and cognitive dissonance theory. Social media makes it easier for users to share their opinions, and users’ web-based obesity-related behaviors have been found to be guided by these theories. In 1995, Rime et al [77] proposed that emotion is a critical motivator in social sharing. The social sharing of emotions has shown that people have an innate need to tell others when they experience an emotionally impactful event [64]. According to So et al [64], a content analysis of frequent retweets about obesity on Twitter, the emotionally evocative tweets, specifically evoking amusement, were the most frequently retweeted. Another theory, cognitive dissonance theory, posits that we experience psychological discomfort when we encounter beliefs that are inconsistent with our own. As a result, people try to reduce this discomfort by exposing themselves to information that helps them resolve cognitive conflicts. A study on Instagram pictures showed that people who reside in high-obesity areas are more willing to post food-related photos than the people in low obesity areas [60]. By understanding the underlying mechanisms of these phenomena, we can better manage obesity-related behaviors.

### Study Year and Region

All the included studies were published between 2011 and 2019, and the number of each study by year is shown in Table 1. This number started to increase in 2014 (n=7) and reached a peak in 2017 (n=14). However, this is partly because our search was conducted in July 2019.

**Table 1:** Number of studies over year.

Year	Studies, n
2011	1
2012	1
2013	1
2014	7
2015	5
2016	12
2017	14
2018	8
2019	1

**Figure 2:**
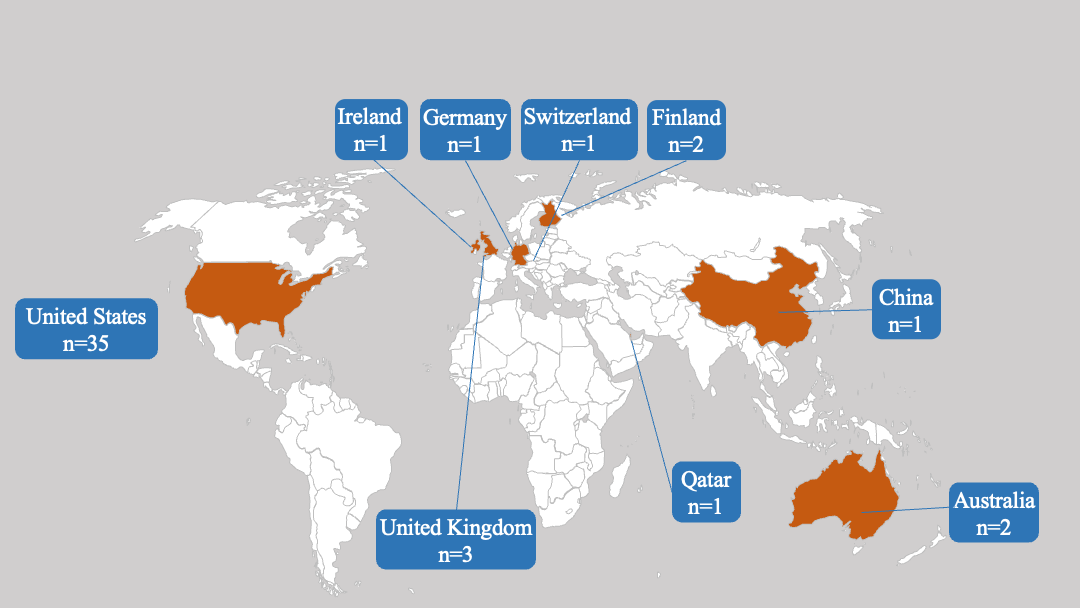
The distribution of study region.

In total, 9 study regions were mentioned, with most of the studies coming from the United States (35/50, 70%). Except for China, Australia, and Qatar, all other regions were located in Europe. The number of studies conducted in other countries is shown in Figure 2. Several groups of users were studied; children, women of childbearing age (eg, pregnant women [78], postpartum women [41], and mothers with newborns [79]), and adults with other relevant illnesses (eg, diabetes [51]) were the 3 leading types of user groups studied. Only the study by Aschbrenner et al [48] focused on adults with obesity and severe mental illness.

### Social Media Platforms and Their Roles

We identified 3 different roles that social media serves in each study. Inspired by a study by Leroux et al [80], three potential roles of social media in obesity-related studies were identified: data collection, intervention pathways, and ancillary resources. Data collection is used to define when social media platforms were only used to collect the data used in the study. The intervention pathway defines social media use as a comprehensive channel in an intervention study. The role of social media includes delivering the message and serving as a web-based communication platform for participants in weight management or loss interventions. We defined ancillary resources as the role when social media is used as an experimental platform. Data were also collected when serving as ancillary resources. The major difference between data collection and ancillary resources is the source of data. If the data were collected for the purpose of analyzing and understanding the data, we defined them as data collection; if the data were generated from the experiment, we defined them as ancillary resources. The type of study can be used to distinguish between intervention pathways and ancillary resources. Social media platforms only serve as an intervention pathway in an intervention study. These categories are mutually exclusive.

We also categorized social media platforms into six different types: microblogging, social networks, weblogs, photo or video sharing, web-based forums, and messaging. Facebook, the largest web-based social network, was the most frequently used (14/50, 28%). Facebook was used the most in intervention studies. Facebook was used as a message delivery channel, in which private groups were introduced as a smaller online support group for participants to share goal-related resources, individual signs of progress, and messages. Only 1 study collected data from Facebook by accessing public Facebook posts. [24]. Similar to Facebook, a Chinese social network platform, WeChat, was used in a study by He et al [46] as the intervention pathway. Twitter allows users to communicate with others from all over the world. Twitter, a microblogging platform, was used to collect data to understand public opinions (11/50, 22%). One study also used Twitter as an ancillary resource (1/50, 2%) to conduct their experiment. For example, May et al [65] created 4 Twitter accounts portraying women (2 obese and 2 normal or overweight) who were interested in weight loss and pretended to behave as regular users. Later, they examined the interaction with other users and mimicked users (eg, follow-back rate). Another study used Twitter as an intervention pathway to involve participants [39]. Few studies have investigated blogs (4/50, 8%), mainly because of the difficulty in extracting meaningful insights from large pieces of text.

Similarly, only 2 studies analyzed clips from YouTube because of the complexity of analyzing videos. Instagram, one of the most prominent photo-sharing platforms, was used in 7 studies. A total of 2 studies used Instagram as an intervention pathway in which participants were asked to upload their meals. In other studies, researchers analyzed public photos to understand public health [59], social movements [55,74], and social sharing behavior [60]. A small number of studies analyzed Twitter and Instagram data with extended data points. For example, researchers combined demographic and geolocation information to better predict the obesity rate of the regions [58]. Reddit has been used in several health-related studies [81]; however, we found only 2 studies using Reddit in our search. Another type of social media is a forum. We identified 3 studies that investigated Yahoo! Answers, a self-developed application (ie, HealthTogether), and another platform not specified in the literature. WhatsApp was chosen as the intervention pathway in 1 study as an alternative to the traditional SMS text messaging method. In this study, group-chat rooms were formed to deliver information and allow participants to communicate on the web. Table 2 presents in the information in a more comprehensive format. The amount of data used in the study and the major study participants are summarized in Table 3.

**Table 2:** A descriptive overview of social media platforms.

Type and platforms		Studies, n	Data collection, n	Intervention pathway, n	Ancillary resource, n
**Microblog (n=14)**					
	Twitter	14	11	2	1
**Social network (n=15)**					
	WeChat	1	0	1	0
	Facebook	14	1	13	0
**Weblog (n=4)**					
	Blogs	4	4	0	0
**Photo or video sharing (n=11)**					
	Instagram	7	5	2	0
	Pinterest	1	1	0	0
	YouTube	3	3	0	0
**Web-based forum (n=11)**					
	Reddit	2	2	0	0
	Yahoo! Answers	1	1	0	0
	Self-developed application	2	0	2	0
	Unknown community	6	3	2	1
**Messaging (n=1)**					
	WhatsApp	1	0	1	0

**Table 3:** Summary of individual studies.

Article	Platform	Region	Group	Data	Web-based social factors	Primary findings	Conceptual
Kang et al [75]	Twitter	United States	UNK^a^	14,317 related tweets	Policy	More negative tweets about the school meal policy have been detected. The main target negative opinions were campaign and food.	Data collection
Fernandez-Luque et al [54]	WhatsApp; Instagram	Qatar	Children	UNK (intervention study)	Gender, geo-cultural factors	More active users tend to have better health outcomes. Females’ engagement with social media is higher. Nutritional advice in weight loss campaigns must consider religious and cultural traditions.	Intervention pathway
Lingetun et al [78]	Blogs	United States	Pregnant women with obesity or overweight	13 internet blogs	Gender	Three main themes of overweight pregnant women’s blogs were identified: pregnancy as an excuse, perspectives on the pregnant body, and becoming a mother.	Data collection
May et al [65]	Twitter	United States	Adults	UNK (experiment study)	Social support, gender, stigma	Investigated follow-back rates. The number of interactions and organic follows did not differ by weight status. Peers interacted more with each other than with professionals. Women need 5 weeks to build a web-based weight loss community on Twitter.	Ancillary resource
Gore et al [57]	Twitter	United States	UNK	More than 25 million tweets	Geo-cultural factors	Geological areas with lower obesity rates (1) have happier tweets and (2) have more frequently discussed food, particularly fruits and vegetables, and physical activities.	Data collection
So et al [64]	Twitter	United States	UNK	200,000 tweets	Social sharing, school environment, obesogenic environment, stigma	Tweets that are emotionally evocative or humorous and express individual-level concerns for obesity were more frequently retweeted than their counterparts.	Data collection
Kent et al [24]	Facebook; Twitter	United States	UNK	291 Facebook posts; 1091 tweets	Obesogenic environment	This study aimed to understand the connection between obesity and cancer from Facebook and Twitter. They found that (1) most tweets focused on an associative or causal link between obesity and cancer, and (2) tweets contained more negative sentiment than Facebook posts.	Data collection
Harris et al [67]	Twitter	United States	Children with obesity	1110 tweets	Source credibility, policy, school environment	This study investigated the communication about obesity on Twitter, and they found that (1) more tweets focused on individual behavior than on policy or environment, and (2) government or educational tweets attract more attention, but the number of these tweets is less.	Data collection
Kuebler et al [53]	Yahoo! Answers	United States	Adults	3926 users’ questions; 300 bullying questions	Gender, stigma, geo-cultural factors	Most women asking whether they were fat or obese were not fat or obese. Users with obesity were significantly more likely to ask for advice about bullying than thinner users. People with obesity who reside in counties with higher BMI may have better physical and mental health than people with obesity who live in counties with lower BMI.	Data collection
Leggatt-Cook and Chamberla [43]	Blogs	United States	Adults	10 blogs	Social support	Weight loss bloggers typically write about daily success and failures, report calorie consumption, and exercise output, and post photographs of their changing bodies.	Data collection
Mejova [62]	Twitter	United States	UNK	1.5 million tweets	Source credibility, stigma	Tweets afflicted with government or institution are likely to be retweeted more. The need to address the quality control of health information on social media is proposed.	Data collection
Munk et al [15]	Instagram	United Kingdom	UNK	82,449 geotagged posts	Obesogenic environment	Sunday night is a good time to post on Instagram. There is no clear difference between thematic communities between high and low BMI areas.	Data collection
Garimella et al [59]	Instagram	United States	UNK	200,000 images	Geo-cultural	Both user-provided and machine-generated image tags provide information that can be used to infer a county’s health statistics.	Data collection
Culotta [58]	Twitter	United States	UNK	1.4-M user profiles and 4.3 M tweets	Geo-cultural	Six of 27 health statistics show a significant correlation with the linguistic analysis of the Twitter activity in the top 100 most populous counties in the United States. Twitter information, together with demographic information, improves the model’s performance.	Data collection
Abbar et al [52]	Twitter	United States	UNK	892,000 tweets	Geo-cultural	The caloric values of the foods mentioned in the tweets were analyzed in relation to the state-wide obesity rate.	Data collection
Weber and Mejova [61]	Twitter	United States	Overweight adults	1339 profile images	Geo-cultural, stigma	User profile pictures could be used to obtain the user’s weight information.	Data collection
Pappa et al [44]	Reddit	United States	UNK	Posts and comments of 107,886 unique users	Social support, gender	The 10 most-discussed semantic topics on posts in the LoseIt Reddit community were related to healthy food, clothing, calorie counting, workouts, looks, habits, support, and unhealthy food.	Data collection
Loh et al [82]	Facebook; Instagram; Twitter	United States	Children	UNK (intervention study)	Social support	The study showed that social media and text messaging were innovative tools that should be included to increase the reach of multilevel community intervention.	Intervention pathway
Ling et al [83]	Facebook	United States	Children	UNK (intervention study)	Social support	Participants in the survey mentioned that they enjoyed the Facebook platform because it provided new recipe and activity ideas and an opportunity to interact with other participants.	Intervention pathway
He et al [46]	WeChat	China	Adults	UNK (intervention study)	Social support	An intervention based on WeChat platform was effective on weight loss only for males. Females show more activities on WeChat, but they lost less weight during the study.	Intervention pathway
Erdem and Sisik [68]	YouTube	United States	Adults	175 videos	Source credibility	There are no significant associations between the number of likes, dislikes, or views and usefulness score. Videos uploaded by medical professionals typically contain more useful information.	Data collection
Jane et al [37]	Facebook	Australia	Adults with obesity or overweight	UNK (intervention study)	Social support	This study shows that participants do not rely on each other in the same way that they would typically rely on their offline social connections. The Facebook group reported the greatest reductions in initial weight compared with the control group, which had no social media components.	Intervention pathway
Fiks et al [79]	Facebook	United States	low-come mothers with a newborn	UNK (intervention study)	Social support	Mothers of the intervention group were significantly less likely to pressure infants to finish food or give cereal in the bottle.	Intervention pathway
Mejova et al [60]	Instagram	United States	UNK	20,848,190 posts	Obesogenic environment, social sharing	There is a link between obesity and the density of fast-food restaurants. Food sharing behavior is higher for high-obesity areas.	Data collection
Cunha et al [42]	Reddit	United States	UNK	70,949 posts and 922,245 comments	Social support	Users receiving feedback on their posts have a higher probability of returning to the community. Returning users who received comments on their posts reported losing more weight.	Data collection
Waring et al [39]	Twitter	United States	Women of childbearing age	UNK (intervention study)	Gender, social support	Women of childbearing age are interested in a weight loss program that was delivered entirely via Twitter.	Intervention pathway
Chomutare et al [47]	UNK	United States	Women with obesity	140 Women with obesity in an internet group	Gender, social support	Women with high web-based participation levels lost more weight than do women with low participation levels.	Data collection
West et al [84]	Facebook	United States	Adults	UNK (intervention study)	Social support	Students maintained their weight, with no significant difference between weight gain prevention intervention group and control group over 9 weeks.	Intervention pathway
Aschbrenner et al [48]	Facebook	United States	Adults with serious mental illness	UNK (intervention study)	Social support	This study shows that weight loss was significantly associated with perceived peer-group support.	Intervention pathway
Merchant et al [40]	Facebook	United States	Adults	UNK (intervention study)	Social support	In a Facebook group that involved weight-loss controlled trial, the following were noted: (1) Polls are the most popular posts followed by photos. (2) Participants visibly engaged with posts less over time. Of participants, 3.4% reported passively engaging with the Facebook page.	Intervention pathway
Chen et al [51]	HealthTogether	Switzerland	Adults	UNK (intervention study)	Social support	Collaborating with buddies to compete in achieving fitness goals in a group was reported as motivating for dyads with strong ties.	Intervention pathway
Phan et al [50]	Web-based social network	United States	Adults	UNK (experiment study)	Obesogenic environment	By incorporating all the human behavior determinants and environmental events, the proposed novel deep learning model achieves more accurate results in predicting the future activity levels of users.	Ancillary resource
Savolainen [45]	Blogs	Finland	UNK	50 blogs	Social support	Blogs provide an emotionally supportive forum that mainly serves to share opinions and information; they were seldom used for seeking information.	Data collection
Church et al [85]	Facebook	UNK	Adults	UNK (intervention study)	Social support	Participants lose weight during the 6-week web-based clinical, emotional freedom techniques course and continue to lose weight in the following year, which indicates the long-term effects.	Intervention pathway
Turner-McGrievy et al [86]	Facebook	United States	Vegan women with polycystic ovary syndrome	UNK (intervention study)	Social support	The study result suggests that engagement with social media may be effective for short-term weight loss among vegan women with PCOS^c^.	Intervention pathway
Lytle et al [49]	Social support website	United States	2-year college students	UNK (intervention study)	Social support, school environment	The social networking encouraged intervention group, and the control group does not have a significant difference in BMI at the end of the 24-month intervention study.	Intervention pathway
Waring et al [41]	Facebook	United States	Postpartum women with overweight or obesity	UNK (intervention study)	Social support	Facebook-based intervention is feasible for overweight and postpartum women with obesity in weight loss. However, research is further needed to determine how to engage participants in social networks better.	Intervention pathway
Basch et al [69]	YouTube	United States	UNK	98 weight loss videos	Source credibility	The number of videos about weight loss on YouTube from professionals is lacking.	Data collection
Webb et al [74]	Instagram	United States	UNK	400 images	Social movement	Health at every size–tagged posts contain more physically active portrayals and weight stigma than do posts from fitspiration-tagged images.	Data collection
Taiminen [87]	Facebook web-based forum	Finland	UNK	UNK (intervention study)	Social support	Active participants in the web-based community showed a more positive perception of achieving their goals, followed instructions more precisely, and perceived to receive more emotional support than participants who are not active in the web-based community.	Intervention pathway
Hales et al [88]	Social POD^b^	United States	Overweight adults	UNK (intervention study)	Social support	The experiment group using a weight-loss mobile app lost significantly more weight than the comparison group.	Intervention pathway
Meitz et al [70]	Facebook	Germany	Children	UNK (intervention study)	Source credibility	In a web-based media-embedded health campaign against childhood obesity, the following were noted: (1) participant’s self-relevance varies based on different source credibility perceptions and (2) provocative messages in the campaign may result in negative persuasion effects.	Intervention pathway
Ghaznavi and Taylor [73]	Twitter and Pinterest	UNK	UNK	300 images	Social movements	The study suggests thinspiration content promotes an objectified, sexual, extremely thin depiction of the thin ideal. Exposure to these contents has the potential harmful effects.	Data collection
Appleton et al [89]	Web-based forums	Australia	UNK	34 discussion threads	Social support	Four major themes were detected in parents’ web-based discussion forums about children obesity: seeking advice, sharing advice, social support, and making a judgment.	Data collection
Karami et al [63]	Twitter	United States	UNK	4.5 million tweets	Social movement	Exercise and obesity, diabetes and obesity, diet, and obesity have a strong correlation with each other. The strongest correlation was found between exercise and obesity.	Data collection
Swindle et al [71]	Facebook	United States	Parents	UNK (intervention study)	School environment	Facebook is a feasible platform to provide nutrition education and facilitate parent’s engagement.	Intervention pathway
De Brún et al [5]	Web-based message boards	Ireland	UNK	2872 obesity-relevant comments	Stigma	The study analyzed obesity-related comments from multi-topic web-based message boards and determined that obesity stigma is pervasive, and the discussion of the issue is highly acceptable.	Data collection
Gregg et al [76]	Web-based forums	United Kingdom	UNK	1704 comments	Policy	The study analyzed associated comments to the United Kingdom government about childhood obesity strategy and determined the comments are largely negative.	Data collection
Atanasova [90]	Blogs	United Kingdom	UNK	343 posts from 6 obesity blogs	Social support	The content of blogs highlighted the conclusion that there are no one-size-fits-all solutions to obesity that work for everyone.	Data collection
Cohen et al [55]	Instagram	UNK	UNK	630 posts	Social movements	Body-positive posts depicted a broad range of body sizes and appearances.	Data collection

^a^UNK: unknown.

^b^POD: social pounds off digitally.

^c^PCOS: polycystic ovary syndrome.

## Discussion

### Principal Findings

In this systematic review, we categorized and identified related the effects of web-based social factors on users’ obesity-related behaviors and evaluated the role of social media. We adopted socioecological model to explain identified web-based social factors at different levels. Moreover, we discussed strategies for preventing obesity by using this socioecological model. We conclude the drawbacks found in the literature and provide suggestions for future studies.

### Socioecological Model

Socioecological models were developed to further the understanding of the dynamic interrelations among various personal and environmental factors [91]. Revised by Bronfenbrenner and Morris [92], the ecological theory of Bronfenbrenner applies socioecological models to human development. The ecological framework identifies five environmental systems with which an individual interacts: microsystem, mesosystem, exosystem, macrosystem, and chronosystem [92]. Since its publication in 1979, the major statement of Bronfenbrenner on the theory of the ecology of human development has shown widespread influence on the way psychologists and others approach the study of human beings and their environments. This socioecological model is proposed to understand how web-based social factors affect behaviors and provide guidance for developing a successful program through web-based social environments.

**Figure 3:**
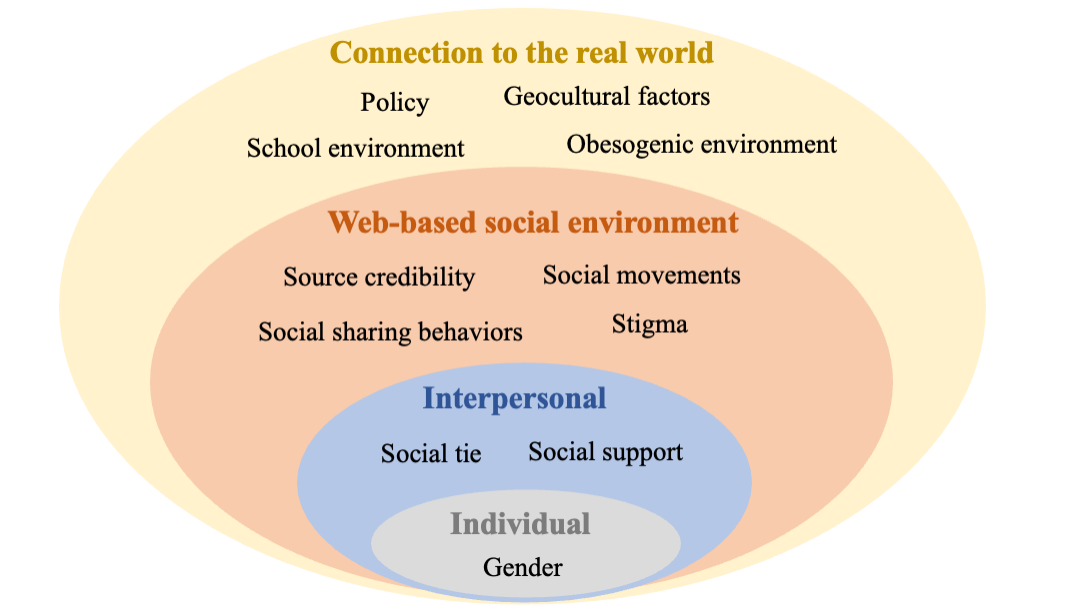
Socioecological model.

We classified these web-based social factors into four levels based on their effects: individual, interpersonal, web-based social environment, and connection to the real world. The proposed socioecological model is shown in Figure 3.

We aimed to identify web-based social factors that can affect users’ obesity-related behaviors. For the web-based environment, we use a 4-level socioecological model to better understand obesity and the effect of potential factors to help users combat obesity. This model considers the complex reciprocity between individual, interpersonal, web-based communities, social media platforms, and connections to the real world. This allowed us to understand the range of factors that potentially affect users’ web-based behavior related to obesity. The overlapping rings in the model illustrate how factors at one level influence factors at another level. Besides helping clarify the effectiveness of these factors, the model also suggests that to intervene in a user’s behavior, it is necessary to act across multiple levels of the model simultaneously.

The first level identifies the personal factors that affect a person’s web-based behavior. The factor identified here was gender. From a biological perspective, women’s bodies are more vulnerable to obesity because women are more likely to store fat because of reproduction [93]. In the web-based environment, female users were more attentive to their shape and figure than were men and were more likely to search and share health information on the web through social media than were men.

The second level explores relationships that may increase or reduce the risk of obesity. People’s close social connections or family members influences impact their behaviors and contribute to their habits. The factors we discovered at this level were social support and web-based social ties. Social support from social media websites has been suggested to be very effective in users’ weight loss experiences, and web-based social ties have been proven to influence a person’s lifestyle behaviors.

The third level examines the web-based social environment in which social relationships occur and the characteristics associated with users’ obesity-related behaviors. The factors that contribute to the web-based environment include source credibility, social movements, social sharing behaviors, and stigma. Social movements, stigma, and source credibility shape users’ behaviors by changing the web-based environment. Exposure to content with obesity-associated stigma has been shown to have negative effects on users’ mental health. Exposure to content from unreliable resources may harm users and further damage their trust in social media platforms. Moreover, exposure to social movements may negatively affect users’ behavior. For example, exposure to content about *thinspiration* would increase a person’s body dissatisfaction and negative mood. Social sharing behaviors change the web-based environment by directly changing users’ sharing behavior. Users prefer sharing emotionally evocative content, especially when consistent with their beliefs.

The fourth level explores the broader societal factors that connect the virtual web-based environment to the real world. Factors at this level include policy, school environment, geo-cultural factors, and an obesogenic environment. The physical environment can inform a virtual web-based environment; for example, the number of fast-food restaurants and food calories mentioned in tweets correlated with the county’s obesity rate. Users’ unsatisfactory opinions of school environments and government policies are found through social media data.

### Preventing Obesity

Reducing the obesity rate requires understanding the factors that influence obesity-related behaviors. This socioecological model helps practitioners develop effective prevention strategies.

Preventive strategies at the individual level promote attitudes, beliefs, and behaviors that combat obesity. The approach may include advocating for males to care more about their weight status and be aware of the importance of having a healthy lifestyle. Female opinion leaders could be encouraged to share healthier lifestyle tips to help women maintain a healthy lifestyle.

A prevention strategy at the interpersonal level may include designing family-focused weight loss programs and supporting users who want to combat obesity to join related web-based groups, share their weight loss or weight management experience on the web, and encourage other users.

Preventive strategies at the social environment level make a point of creating a healthier internet social environment; for example, improving the source credibility level by monitoring illegal advertisements and advocating professional organizations to post strategies combating obesity, coping with stigma issues by adopting state-of-the-art natural language processing technologies to remove stigmatized posts from social media, supervising the web-based environment by detecting major social movements that intersect with obesity, and building users’ healthy life beliefs by encouraging positive social sharing on social media. Emotionally evocative posts are more easily accepted, depending on users’ social sharing behaviors.

Preventive strategies related to the real-world level emphasize building a healthy societal environment and establishing a good connection between the web-based environment and real society. Social policies from the government that lead a healthy lifestyle, a good school environment that provides children with a balanced diet, and an antiobesogenic environment can help maintain a healthy societal environment.

### Data Variety

We observed some drawbacks in the included studies. The data variety needs to be expanded in future studies. First, the limitations of each social media platform should be considered. Although Twitter is one of the biggest web-based social network platforms, it may not serve as the best channel for collecting data and studying obesity-related topics. Twitter is a platform that makes data publicly available. Because of privacy and stigma concerns, some users may refuse to share confidential data on Twitter [82]. Comparing 3 major social media platforms (Facebook, Instagram, and Twitter) in a childhood obesity prevention intervention by Loh et al [82], Facebook—allowing more comprehensive communication and longer and more frequent posts than the other 2 social media platforms—was found to have the highest fidelity and engagement. In contrast, Twitter has the least engagement and fidelity [82].

Second, a large amount of image data is an emerging resource. We discovered that textual data were the leading type in previous studies, and a large amount of media-syncretic image data was dismissed. A study from Mejova et al [60] analyzed picture tags and images. Textual data, along with their associated features (eg, image, link, and user profile), could provide more insight. Third, the regions of the studies were too limited. The prevalence of obesity is also high in other areas of the world, such as Mexico [94]. Given the cultural differences, it is meaningful to understand the social elements of other areas. Another finding was that the data collected through experiments were not analyzed. Almost all intervention studies encouraged participants to interact with others on social media platforms; the efficacy of these social components did not receive adequate study. Only 2 studies performed quantitative analysis on user interaction behavior (eg, how many posts the user submits every day) [46,51] in an online support group. No qualitative analysis was employed on the textual data collected in the study, which could give us a clue as to how factors affect people’s weight loss experience.

Finally, the data quantity in many studies was insufficient, considering the large number of people with obesity. For example, studies using blog data to perform qualitative analyses used just 10 [43] and 13 [78] blogs. Manually conducting a thematic analysis is indeed labor-intensive; however, with the development of deep learning, pretrained language models could be effectively employed to analyze large amounts of data.

### Limitations

Our systematic review has some limitations. First, we only used three databases (PubMed, ACM, and ScienceDirect) in our study. If other databases, such as PsycINFO, Embase, and Scopus, were included in the study, we might have had additional and possibly different findings. Second, the MeSH term *social media* was created in 2012 [34]; thus, our study did not include studies published before 2012. This may have skewed the results. Future studies should consider a broader search strategy for more comprehensive results. Third, many studies did not include a particular type of social factor and how those factors affected users; thus, the analysis of social factors was not sufficient. Finally, further discussion on the quality of study design, types of bias, and other limitations of the investigated studies can bolster our findings.

### Conclusions

We provided a comprehensive review of social media in relation to understanding obesity and isolating web-based social factors, including platforms, data, and study results. We proposed a 4-level socioecological model to explain the dynamic interrelationships among users’ obesity-related behaviors, personal characteristics, users’ interpersonal connections, web-based social environments, and the real world. Understanding the potential role of these factors will benefit us in several aspects: understanding users’ web-based social behaviors concerning obesity, calibrating web-based social factors for weight management intervention studies, and disseminating educational information to the public.

## Abbreviations

ACM: Association for Computing Machinery
HAES: health at every size
MeSH: Medical Subject Heading
